# Mesalazine-Induced Myopericarditis in a Patient with a Recent Diagnosis of Crohn's Disease: Apropos of a Case

**DOI:** 10.1155/2015/728310

**Published:** 2015-07-21

**Authors:** Michele Sorleto, Stefanie Dürrwald, Marcus Wiemer

**Affiliations:** Department of Cardiology, Johannes-Wesling-Klinikum Minden, Hans-Nolte-Strasse 1, 32429 Minden, Germany

## Abstract

Mesalazine- (5-aminosalicylic acid-) containing products are a well-known treatment for inflammatory bowel disease, often as first line. Myocarditis is recognized as a very rare possible side effect of this drug treatment. We present a case of mesalazine-induced myopericarditis that was successfully improved by immediate cessation of the medication.

## 1. Introduction

Mesalazine- (5-aminosalicylic acid-) containing products are a well-known treatment for inflammatory bowel disease, often as first line. Hypersensitive reactions to mesalazine have been reported and include mostly gastrointestinal upset and headaches [1]. Although the prevalence is very rare, myocarditis is recognized as a possible side effect of these medications [2–4].

## 2. Case Presentation

**Figure 1:**
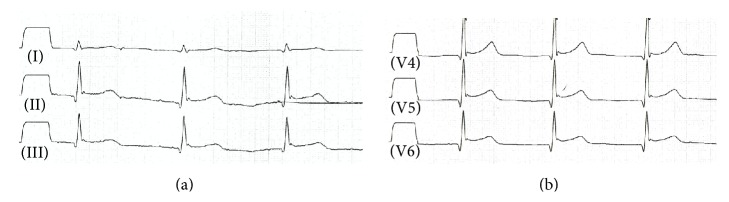
Initial 12-lead electrocardiogram: sinus rhythm and ST-segment elevation with upward concavity in leads II, III, aVF, and V4–V6.

Here, we present a case of mesalazine-induced myopericarditis in an 18-year-old male patient with a recent diagnosis of Crohn's disease on treatment with mesalazine and low dosages of prednisone. The young patient was admitted to our hospital via emergency room with retrosternal chest pain spreading to the left arm for several hours. The inflammatory bowel disease was well controlled and there was no reported infection in the last 6 months. The physical examination was normal and there were no signs of hemodynamic instability or heart failure. Initial 12-lead electrocardiogram showed a sinus rhythm and ST-segment elevation with upward concavity in leads II, III, aVF, and V4–V6 (Figure 1). The laboratory tests revealed elevated cardiac biomarkers more than 3 times upper limits (peak troponin hs 1158 pg/mL and peak total Creatin kinase 547 U/L), a C-reactive protein concentration of 97.4 mg/L, and other blood tests within normal parameters (Table 1). A coronary angiography was performed and demonstrated normal epicardial vessels. A cardiovascular magnetic resonance imaging confirmed the coronary angiography result and also there was no hint for a myocardial infarction. However, it showed a late gadolinium enhancement and a myopericardial oedema. As myopericarditis was suspected as an adverse reaction associated with treatment with mesalazine, the drug was immediately stopped with maintaining low dosages of prednisone and metamizol. After that, the retrosternal chest pain has gone and the cardiac biomarkers decreased to normal value. The abnormal ECG findings have been gradually normalized within 7 days. Echocardiographic measurements were within the normal limits. After 8 days the patient was discharged in a good condition to home.

## 3. Discussion

Cardiac involvement can be associated with Crohn's disease as an extraintestinal manifestation of the inflammatory bowel disease. It has also been described as a side effect of the treatment of inflammatory bowel disease with mesalazine. Myocarditis is recognized as a very rare possible side effect of mesalazine- (5-aminosalicylic acid-) containing products, generally occurring 2–4 weeks after the initial exposure to the drug. Several cases were described in the literature. Resolution of symptoms has occurred in all reported cases within one week after drug discontinuation [5–8]. Although the precise pathophysiology of mesalazine-induced myopericarditis is poorly understood, it is thought to be a hypersensitivity reaction rather than a cytotoxic effect. A proposed mechanism is humoral-mediated hypersensitivity in which antibodies formed against mesalamine cross-react with cardiac tissue causing inflammation [9]. An eosinophilic infiltration of the myocardium on endomyocardial biopsy has been described, which seems to confirm the link between mesalazine and hypersensitivity [10]. In our case the patient has been treated for 6 weeks with mesalazine containing oral medication, before the drug induced cardiovascular toxicity. Even though myopericarditis is an uncommon adverse reaction to mesalazine, it seemed to be attributed in our young patient during drug treatment. Within 48 hours after discontinuation of the medication, the sick feeling person evolved well and remained asymptomatic, with decreasing cardiac biomarkers. Considering this clinical course plus the fact that there was no previous infection, the immediate drug withdrawal achieved clinical improvement. Therefore, myopericarditis was considered to be the most likely diagnosis. In conclusion, we present a case of mesalazine-induced myopericarditis that was successfully improved by immediate cessation of the medication. In our case, the onset and resolution of symptoms within one week after drug discontinuation were similar to those reported in literature [3, 4, 7, 8]. This case identifies the importance of prompt evaluation, diagnosis, and eliciting a thorough medical history when new medications are started. The risk of mesalamine-induced cardiac involvement needs to be considered in patients with inflammatory bowel disease after recent exposure to the drug. It is suggested that every patient with chest pain during mesalamine therapy should be evaluated via cardiac enzymes, electrocardiogram, echocardiogram, and possibly cardiovascular magnetic resonance imaging to rule out this rare drug-induced disorder.

## Figures and Tables

**Table 1:** Initial and follow-up biochemistry values.

Laboratory chemical analysis	Day 1 4:50	Day 1 7:40	Day 4 9:30	Day 7 9:30
Troponin hs (pg/mL)	822.6	1158.0	149.3	9.3
Creatin kinase (U/L)	432	547	43	39
C-reactive protein (mg/L)	97.4		58.2	11.1
Leukocyte (G/L)	14.7		12.1	8.0
